# Diagnosis of Neurological Infections in Pediatric Patients from Cell-Free DNA Specimens by Using Metagenomic Next-Generation Sequencing

**DOI:** 10.1128/spectrum.02530-22

**Published:** 2023-01-18

**Authors:** Xia Li, Le Yang, Dongjing Li, Xuying Yang, Zhijing Wang, Mengyi Chen, Fang Wu, Xiangjun Dou, Mengmeng Niu, HongYan Qi, Ting Deng, Han Xia, Dong Wang

**Affiliations:** a Department of Pediatric Neurology, Xi’an Children’s hospital, Xi’an City, Shaanxi Province, China; b Department of Scientific Affaires, Hugobiotech Co., Ltd., Beijing, China; National Institute of Allergy and Infectious Diseases

**Keywords:** mNGS, pediatric, cell-free DNA, whole-cell DNA, neurological infections

## Abstract

Central nervous system (CNS) infections can cause significant morbidity and mortality, especially in children. Rapid and accurate pathogenic detection in suspected CNS infections is essential for disease control at the early stage of infection. To evaluate the performance of metagenomic next-generation sequencing (mNGS) of cell-free DNA (cfDNA) in cerebrospinal fluid (CSF) in pediatric patients, we retrospectively analyzed the efficiency of cfDNA mNGS in children with CNS infections (*n* = 257) or noninfectious neurological disorders (*n* = 81). The CSF samples of 124 random subjects were used to evaluate the accuracy between mNGS of cfDNA and whole-cell DNA (wcDNA). In total, cfDNA mNGS detected a wide range of microbes with a detection rate of 71.0%, and the sensitivity and total coincidence rate with clinical diagnosis reached 68.9% and 67.5%, respectively. Compared with wcDNA mNGS, cfDNA mNGS had a higher efficacy in detecting viruses (66 versus 13) and Mycobacterium (7 versus 1), with significantly higher reads per million. The dominant causative pathogens were bacteria and viruses in CNS infections, but these presented with different pathogen spectra in different age categories. The best timing for the mNGS test ranged from 1 to 6 days after the start of anti-infection therapy, and the earlier mNGS started, the better was identification of bacterial CNS infections. This study emphasized that cfDNA mNGS had overall superiority to conventional methods on causative pathogen detection in pediatric CNS infections, and it was even better than wcDNA mNGS. Furthermore, research needs to be better validated in large-scale clinical trials to improve the clinical applications of cfDNA mNGS.

**IMPORTANCE** Our study emphasized that cfDNA mNGS had overall superiority to conventional methods on causative pathogen detection in CNS-infected children, and it was even better than wcDNA mNGS. cfDNA mNGS detected a wide range of pathogens with a high total coincidence rate (67.5%) against clinical diagnosis. The best timing for cfDNA mNGS detection ranged from 1 to 6 days, rather than 0 days, after the start of empirical anti-infection therapy. The earlier mNGS started, the better the identifications of bacterial CNS infections. To the best of our knowledge, this research is the first report evaluating the clinical utility of mNGS with different methods (cfDNA versus wcDNA) of extracting DNA from CSF specimens in diagnosing pediatric CNS infections. Meanwhile, this is the largest cohort study that has evaluated the performance of mNGS using cfDNA from CSF specimens in pediatric patients with CNS infections.

## INTRODUCTION

Central nervous system (CNS) infection presents a severe life-threatening condition that can disseminate and lead to inflammatory disorders, such as meningitis, encephalitis, meningoencephalitis, and transverse myelitis (1), causing significant morbidity and mortality (2), especially in pediatric patients (3). All the microbial pathogen types are able to cause a variety of CNS infections (4), of which bacteria and viruses are more frequent in children (5, 6). A multinational study of community-acquired CNS infections from 37 referral centers in 20 countries found that the most frequent infecting pathogens were Streptococcus pneumoniae (8%) and Mycobacterium tuberculosis (5.9%) (7). A hospital-based study in Beijing reported that approximately 39% of 1,212 children admitted for tuberculosis treatment had tuberculous meningitis (8). Other common organisms that cause childhood meningitis are Haemophilus influenzae, Neisseria meningitidis, and Streptococcus agalactiae (5). In addition, several viruses, such as Japanese encephalitis virus, enteroviruses (EVs), herpes simplex viruses (HSVs), arboviruses, and influenza viruses, can cause CNS infections in sporadic, endemic, epidemic, or pandemic patterns (9).

The positive rate in cerebrospinal fluid (CSF) culture is less than 20% (10), while other approaches, such as serological antibody tests and PCR assays (11), require several prior assumptions that limit their application. In fact, studies have shown that an etiological diagnosis is not obtained in 40 to 50% of CNS infections when using these conventional methods (3, 12). The undiagnosed proportion of pediatric CNS infections is even greater due to nonspecific symptoms and physical signs (3, 13), which may lead to the inappropriate usage of antibiotics or even adverse progress (14). Therefore, rapid and accurate detection of pathogens is essential for disease control at the early stage of suspected CNS infections (15). Metagenomic next-generation sequencing (mNGS) (16, 17) has been proven to detect nearly all known pathogens from clinical samples (18, 19). Using cell-free DNA (cfDNA) to perform mNGS has also proven to be a promising tool in detecting pathogens from body fluids, with higher sensitivities (75 to 91%) and specificities (81 to 100%) (20) for extracellular pathogens as well as intracellular pathogens (17).

A growing body of evidence suggests that mNGS might improve the diagnostic efficiency of pathogen identification in pediatric CNS infections (21–23), among which the reported sensitivity of cfDNA mNGS has reached up to 90% (24, 25), but most of these were case reports or small cohort studies with limited reference values. Human DNA contamination remains a major challenge to detect pathogens by mNGS, as a high background of host DNA greatly reduces the sequence coverage of the pathogens in most clinical specimens (26). The differential lysis method can filter human DNA from high human DNA background specimens and increase the pathogen DNA ratio, but at the expense of some viruses, parasites, and bacteria (20, 27). Compared to cfDNA mNGS, although the mNGS of whole-cell DNA (wcDNA) obtained from the differential lysis method can detect more reads per million (RPM) of Cryptococcus neoformans, its detection of trace M. tuberculosis was hindered (28). Moreover, the detection rate of mNGS directly using the wcDNA of CSF samples without differential lysis was only ~50% (29). On the other hand, the locations of proliferation and infection between intracellular and extracellular pathogens also exhibit long-term contradictions, and the DNA of intracellular pathogens can exist in body fluids in the form of cfDNA (30). Therefore, we hypothesized that the performance of cfDNA mNGS is better than that of wcDNA mNGS in CNS infections. A recent study showed that the sensitivity of cfDNA mNGS was higher than that with wcDNA when using CSF samples in adults, especially for viral and mycobacterial pathogens (31), but evidence for children with CNS infections is still lacking. Moreover, there is little published information on the contribution of mNGS to noninfectious disease pathologies.

Hence, we retrospectively analyzed the performance and efficiency of cfDNA mNGS in pediatric patients with CNS infections (*n* = 257) and noninfectious neurological disorders (*n* = 81), and pathogen profiles were also summarized. To evaluate the accuracy between cfDNA and wcDNA mNGS, we randomly selected 124 CSF samples from all the enrolled subjects to perform mNGS tests using both cfDNA and wcDNA simultaneously.

## RESULTS

### Clinical conditions and symptoms.

A total of 338 participants were retrospectively enrolled, with a median age of 3.0 years (range, 0.1 to 14.8 years), of whom 199 (58.8%) were male. In the entire group, 257 pediatric patients (74.3%) were diagnosed with CNS infections, including bacterial meningitis in 132 patients (38.2%), viral encephalitis or meningoencephalitis in 110 (31.8%), and other CNS infections in 15 (4.3%). Additionally, 81 pediatric patients were diagnosed with noninfectious neurological diseases, such as autoimmune encephalitis, toxic encephalopathy, and epilepsy. Table 1 presents the demographic information, clinical symptoms, and clinical diagnoses of these pediatric patients. Most children reported at least one prodromal symptom, including 262 cases (77.5%) of fever, 128 (37.9%) of vomiting, 80 (23.7%) of headaches, 143 (42.3%) of convulsion, 195 (57.7%) of focal neurological dysfunction, 156 (46.2%) of meningeal irritation, and so on. A total of 235 (67.9%) and 123 (36.4%) children had magnetic resonance imaging (MRI) and electroencephalogram (EEG) abnormalities, respectively.

**TABLE 1:** Clinical characteristics of the 338 pediatric patients

Characteristic	No. (%)
Patient demographics (*n* = 338)	
Gender	
Male	199 (58.8)
Female	139 (41.1)
Age	3.0 yrs (IQR, 0.5–7.8)
Clinical diagnosis	
CNS infection	
Bacterial meningitis	132 (38.2)
Viral encephalitis/meningoencephalitis	110 (31.8)
Mycobacterial meningitis	8 (2.3)
Mycoplasma encephalitis	4 (1.2)
Fungal meningitis	2 (0.6)
Uncertain CNS infectious disease	1 (0.3)
Noninfectious encephalopathy	
Autoimmune encephalitis	28 (34.6)
Infectious toxic encephalopathy	11 (13.6)
Epilepsy	9 (11.1)
Other disorders	33 (40.7)
Concomitant disease	
Pulmonary infection	92 (26.6)
Hyponatremia	18 (5.2)
Myelitis	16 (4.6)
Clinical symptoms	
Fever	262 (77.5)
Vomiting	128 (37.9)
Headaches	80 (23.7)
Convulsion	143 (42.3)
Spasm	142 (42.0)
Focal neurological dysfunction	199 (57.5)
Meningeal irritation	156 (45.1)
Conscious disturbance	85 (25.1)
Dystonia	74 (21.9)
Abnormal MRI	235 (67.9)
Abnormal EEG	123 (36.4)
Abnormal parameters of CSF examinations	
Increased CSF leukocyte counts	209 (61.8)
Elevated CSF protein level (>0.4 g/liter)	173 (51.2)
Decreased CSF glucose level (<2.8 nmol/liter)	112 (33.1)

### Clinical metagenomes of cfDNA versus wcDNA for CNS infections.

**FIG 1:**
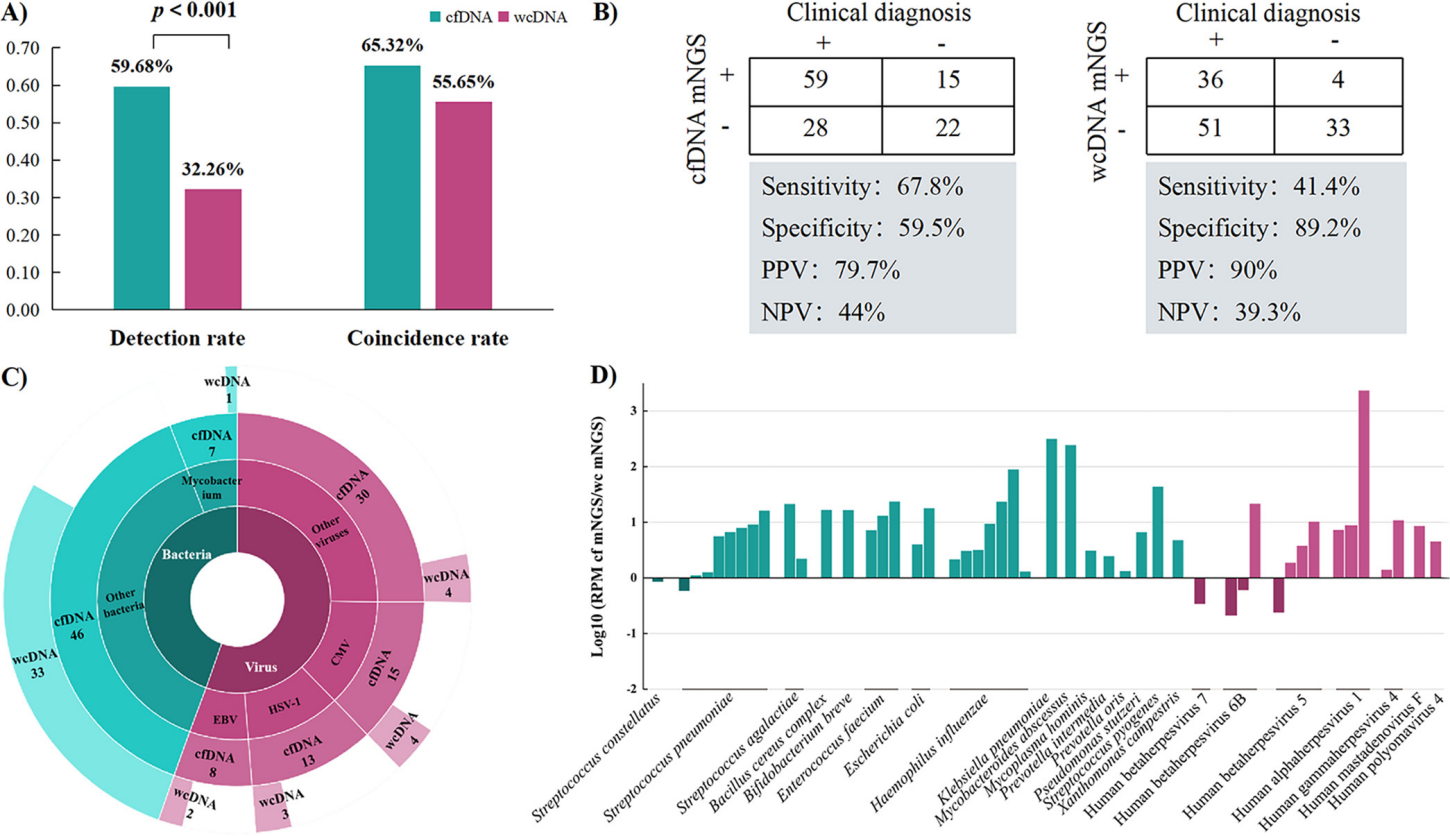
Comparison between cfDNA and wcDNA mNGS for clinical utility in CNS infections. (A) Comparison of detection rates and coincidence rates. (B) Contingency tables for clinical diagnosis with cfDNA mNGS and wcDNA mNGS sets, respectively. (C) Advantages of cfDNA mNGS over wcDNA mNGS. (D) Ratio of RPM for pathogens of the same patients detected by both strategies.

To compare the clinical utility of different extraction methods for microbial DNA, we randomly selected 124 CSF samples from all the enrolled subjects to perform mNGS tests using both cfDNA and wcDNA simultaneously. Our data showed that the detection rate of cfDNA mNGS (59.68%, 74/124) was significantly higher than that of wcDNA mNGS (32.26%, 40/124) (*P* < 0.001, chi-square test) (Fig. 1A), especially for viral and intracellular microbes. In addition, the total coincidence rate (TCR) and sensitivity of cfDNA mNGS were much higher than those of wcDNA mNGS (65.32% versus 55.65%, *P* = 0.119, chi-square test; and 67.8% versus 41.4%, *P* < 0.01, chi-square test, respectively) (Fig. 1B). Although the performance of wcDNA mNGS (*n* = 34) for detecting bacteria was close to that of cfDNA mNGS (*n* = 46), Mycobacterium in 85.7% of patients (6/7) could only be detected by cfDNA mNGS (Fig. 1C), and the remaining bacteria detected only by cfDNA mNGS in the 19 patients were Rickettsia felis, *Enterococcus* spp., and Mycoplasma hominis. Higher efficacy of cfDNA mNGS could also be found in detecting viruses (66 versus 13), including cytomegalovirus (CMV; 15 versus 4), human alphaherpesvirus 1 (HSV-1; 13 versus 3), and Epstein-Barr virus (EBV; 8 versus 2) (Fig. 1C). Additionally, for microbes from the same patients detected by both strategies, the detected RPM by cfDNA mNGS was significantly higher than that by wcDNA mNGS (Fig. 1D). These results revealed that the accuracy and sensitivity of cfDNA mNGS were better than those of wcDNA mNGS in the diagnosis of CNS infections, especially for viral and mycobacterial CNS infections.

### Performance and efficiency of cfDNA mNGS.

**FIG 2:**
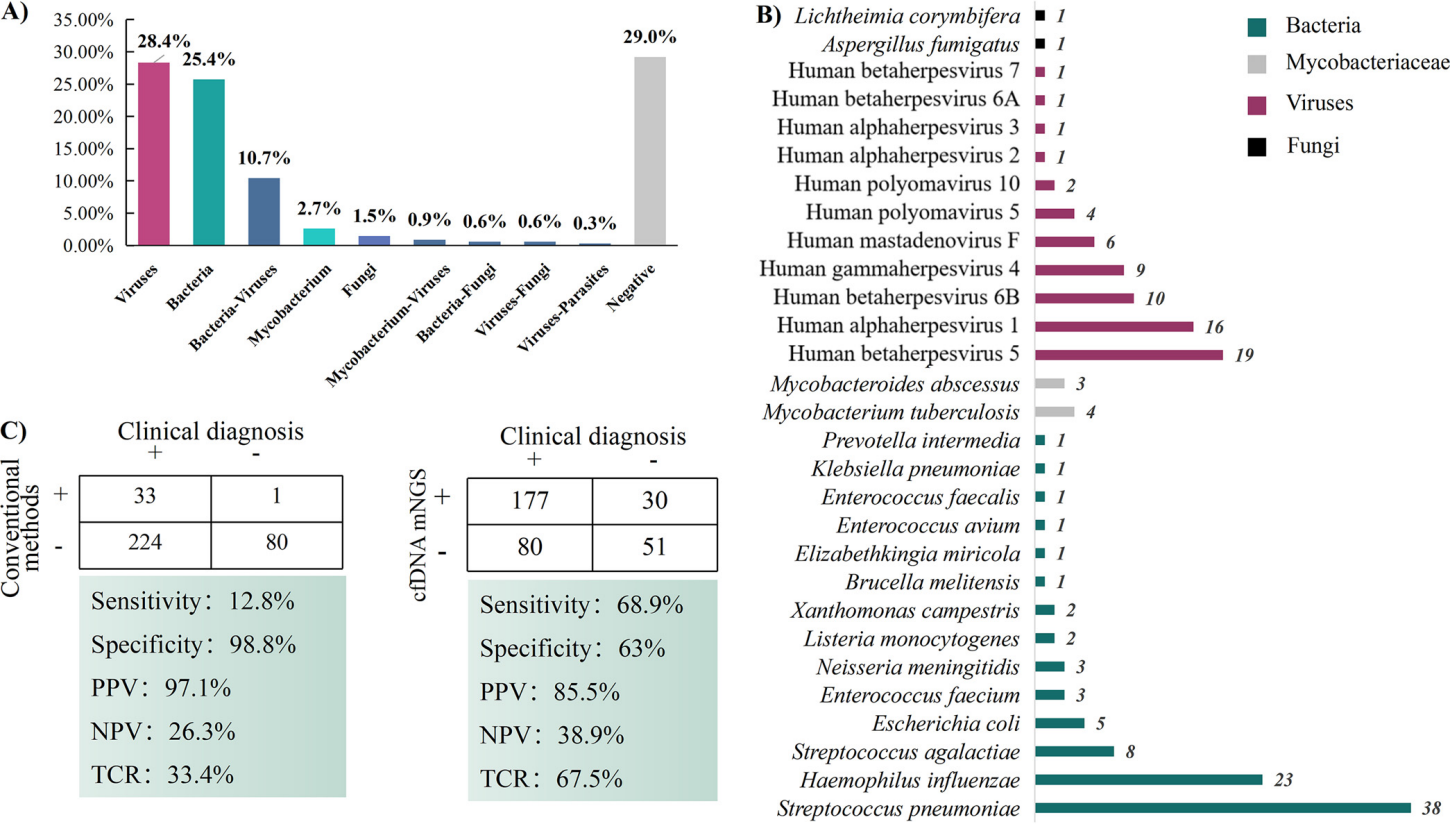
Performance and efficiency of cfDNA mNGS. (A) Potential pathogens detected by cfDNA mNGS. (B) Distribution of predominant pathogens in CSF samples. (C) Diagnostic efficiency by mNGS and conventional methods based on the clinical diagnosis.

The positive rate of cfDNA mNGS detection was 71.0% (240/338), and two or more microbes were detected in 44 patients (Fig. 2A). Bacteria (*n* = 43) were the most common microbes detected in 53.3% (128/240) of pediatric patients, and 33 bacterial species were consistent with clinical diagnosis, including S. pneumoniae, H. influenzae, S. agalactiae, Escherichia coli, and M. tuberculosis (Fig. 2B). Twenty-seven viruses were detected in 44.2% (106/240) of patients, and at the top of the list of 20 clinically relevant viruses was CMV, followed by HSV-1, human betaherpesvirus 6B (HHV-6B), and EBV (Fig. 2B). Additionally, for only two of seven fungi, Aspergillus fumigatus and Lichtheimia corymbifera, were identifications in agreement with clinical diagnosis. Conventional methods, including CSF cultures, smears, and viral serologies, only identified pathogens in 11.8% (40/338) of the CSF samples, of which 23 CSF cultures were positive. Rare and intracellular pathogens, such as N. meningitidis and M. tuberculosis, were detected solely by mNGS.

Among the 257 children with CNS infections, 207 patients had positive mNGS results, with 85.5% (177/207) accuracy. Further analysis of 81 noninfectious neurological diseases revealed that the true-negative (TN) rate of mNGS was 63% (51/81) (Fig. 2C). Considering the clinical diagnosis as a unified standard (Fig. 2C), the sensitivity and specificity of cfDNA mNGS were found to be 68.9% and 63.0%, respectively, whereas those of conventional methods were 12.8% and 98.8%, respectively. In addition, 67.5% of the mNGS results were consistent with clinical diagnosis, whereas conventional methods obtained a concordance rate of 33.4%. These data demonstrated that cfDNA mNGS had significantly greater sensitivity and coincidence for detecting potential pathogens than conventional methods and was superior in diagnosing suspected CSF infections.

### Pathogen profiles and etiology of pediatric CNS infections.

**FIG 3:**
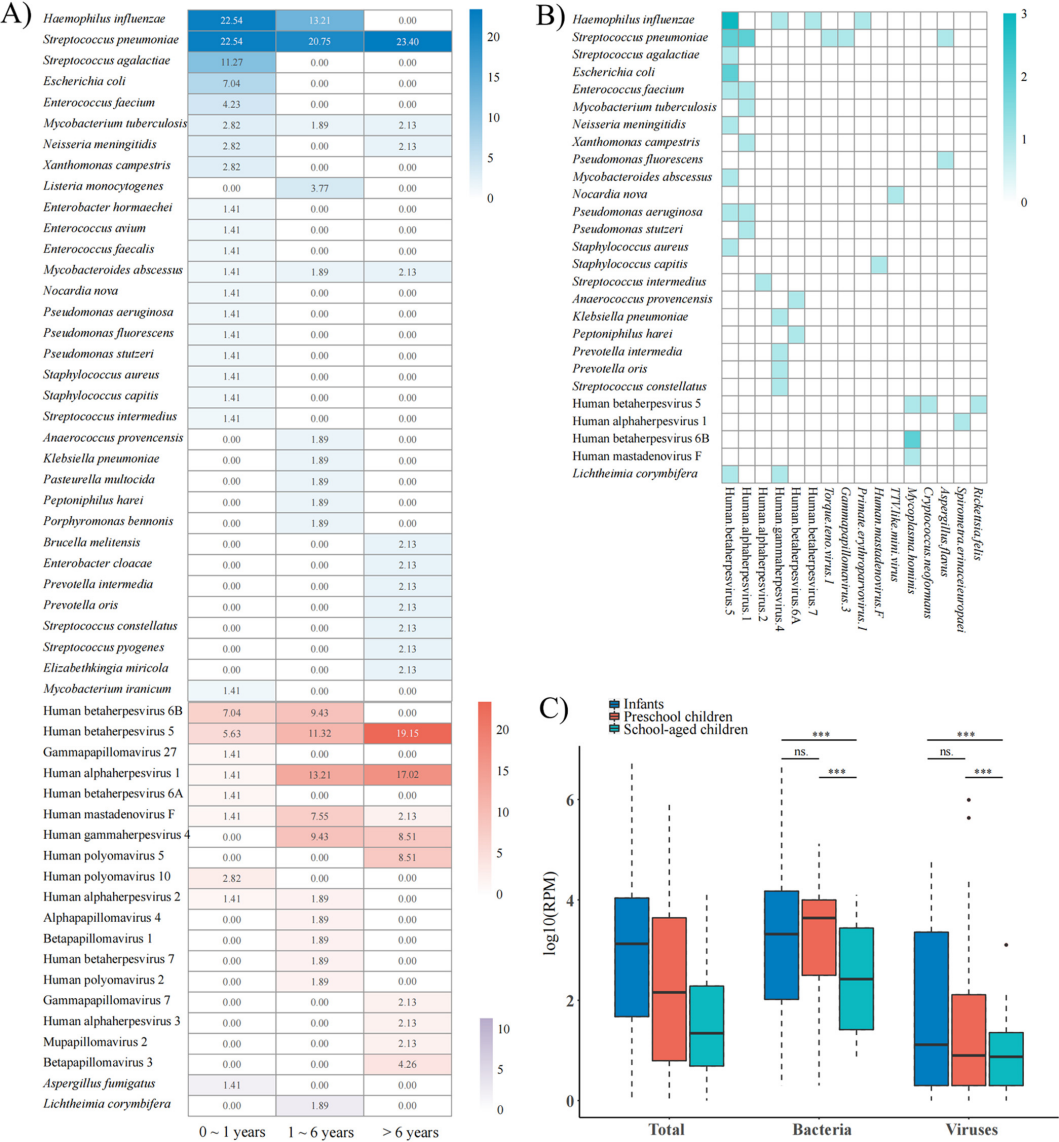
Pathogen profiles and statistics of CNS infections in pediatric patients. (A) Pathogen profiles of mNGS for different age categories. (B) All of the clinically irrelevant microbes detected in bacterial, viral, and fungal infections. The clinically irrelevant microbes are presented on the *x* axis, and the causative pathogens in different types of infections are presented on the *y* axis. (C) Boxplot statistics for the detected RPM in different age categories.

In total, 29 (54.7%), 21 (39.6%), and 20 (37.7%) of 53 causative pathogens were detected in 126 infants (0 to 1 years), 97 preschool children (1 to 6 years), and 115 school-aged children (>6 years), respectively (Fig. 3A). The main causative pathogens identified in bacterial infections were S. pneumoniae, with contributions of 22.54%, 20.75%, and 23.4% in infants, preschool children, and school-aged children, respectively. However, H. influenzae was only identified in infants and preschool children, with contributions of 22.54% and 13.21%, respectively. The numbers of bacterial species were the highest (*n* = 20) among infants, and rare pathogens were more likely to be detected in school-aged children. The proportions of viral infections in the three age categories were 22.54%, 60.38%, and 68.09%, respectively. CMV, HSV-1, and human mastadenovirus F were identified in all age categories, while 10 other viruses were detected in children of all ages except infants. Additionally, among the 44 patients in which two or more microbes were detected, CMV, HSV-1, EBV, etc., were also detected with a wide range of RPM (1 to 500) in 33 bacterial infections (Fig. 3B). However, we found that bacteria or fungi were rarely detected in viral infections. Moreover, we found that the detected RPM of bacteria and viruses in school-aged children were significantly lower than that in children in the other two age categories (Fig. 3C).

### Relationship between cfDNA mNGS and clinical management.

**FIG 4:**
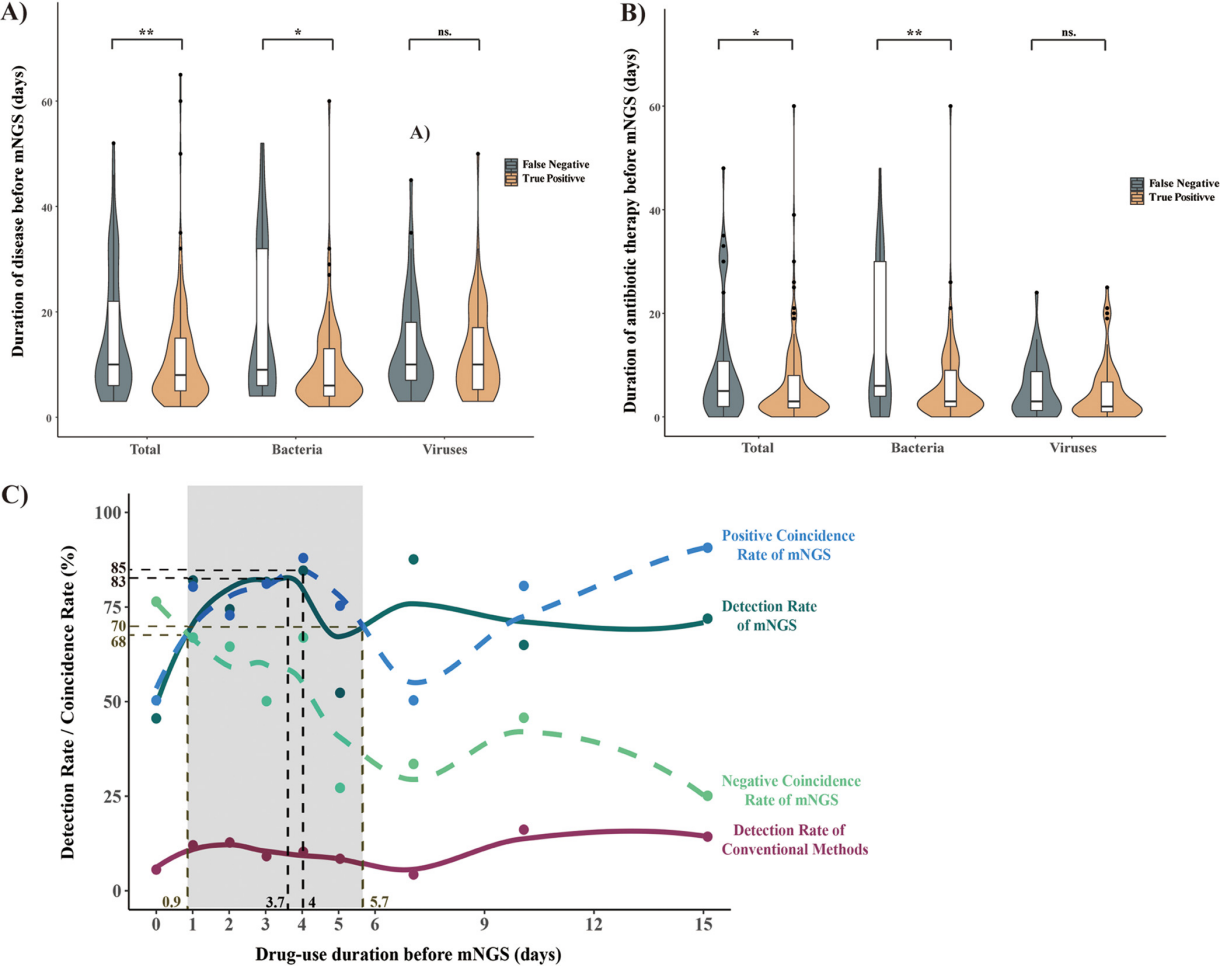
Relationship between mNGS and clinical risk factors. (A) Correlation between sampling time and cfDNA mNGS results. (B) Correlation between medication time and cfDNA mNGS results. (C) Positive coincidence rate and detection rate of cfDNA mNGS changes with the duration of empirical therapy before mNGS tests. Teal circles represent detection rate of mNGS with empirical therapy for 0, 1, 2, 3, 4 to 5, 6 to 9, or 10 to 15 days. The teal curve represents detection rates of mNGS fit using local polynomial regression fitting. Purple circles and curve represent detection rates of conventional methods. Blue circles and dashed curve represent fit positive coincidence rate of mNGS. Green circles and dashed curve represent fit negative coincidence rate of mNGS.

All pediatric patients were treated with empirical therapy at the onset of admission, and the duration before mNGS tests ranged from 0 to 60 days. To determine the best time to perform mNGS in CNS infections, the sampling times of CSF specimens for mNGS after disease onset and medication time of the TP and FP results were compared. Our data indicated that there was a significant difference (5.9 ± 7.5 versus 9.1 ± 10.5; *P* = 0.02) in the medication time of the two groups (Table 1). Furthermore, there was a significant correlation between sampling-medication time and cfDNA mNGS results in the bacterial infection subgroup. However, the time showed no significance in the viral infection subgroup (Fig. 4A, B). Based on local polynomial regression, we observed that the detection and compliance ratios of cfDNA mNGS decreased when the antibiotics were administered for longer than 4 days (Fig. 4C). The positive coincidence rate (PCR) of cfDNA mNGS peaked at 85% on day 4 and remained above 70% from 0.9 to 5.7 days. Then, the PCR fell to the lowest point on day 8, but the curve remained higher than the rate of the conventional methods from day 1. Consequently, we proposed that cfDNA mNGS tests should be performed during a duration of 1 to 6 days, and the earlier that mNGS is started, the better bacterial CNS infections can be identified in pediatric patients.

## DISCUSSION

To the best of our knowledge, this research is the largest cohort study evaluating the performance of cfDNA mNGS in the diagnosis of pediatric CNS infections. Our data imply that cfDNA mNGS using CSF specimens is a practical tool for the diagnostic evaluation of children with neurological infection but may not be appropriate to rule out noninfectious disorders. The bacterial and viral pathogens presented with different pathogen profiles in different age categories. The best timing for cfDNA mNGS ranged from 1 to 6 days after the start of anti-infection therapy, and the earlier that mNGS was started, the better the identification of bacterial CNS infections in pediatric patients.

CNS infection is a severe disease with high morbidity and disability, while limited sample volumes and low detection efficiency (32) hinder the comprehensive detection of such pathogens using conventional methods. Meanwhile, the detection performance of conventional methods was sensitive to anti-infective therapy, which had a hysteresis effect on mNGS results (29, 33). Molecular multiplex CNS infection diagnosis panels have been developed and present several absorbing features, including ease of use and low turnaround time (34). A fully automated multiplex PCR, the BioFire FilmArray meningitis/encephalitis (FA-ME) panel, which was the first nucleic acid-based CSF test cleared by the U.S. Food and Drug Administration in October 2015, detects 14 common pathogens simultaneously in an hour (35). Although FA-ME testing is feasible, timely, and convenient, studies investigating the performance and clinical applications of FA-ME are scarce, especially for pediatric patients (36). In our cohort, more than 40 pathogens, including M. tuberculosis and EBV, were not included in the FA-ME panel, indicating that commercially available kits are not the optimal method for use in northwest China. Studies have documented that cfDNA mNGS has a better performance in detecting pathogens in body liquids (37), plays a unique role in identifying rare pathogens in CSF specimens (20, 33, 38), and displays high sensitivity that is less affected by the human background (39). Our current study confirmed that cfDNA mNGS was superior to conventional methods in pathogen detection, with a high TCR (67.5%) and sensitivity (68.9%), which presented similar performance in previous studies (40). However, one of the shortcomings of mNGS is still its cost compared to conventional methods and other commercially available molecular diagnostic tools. For full potential of cfDNA mNGS to be realized, the cost of the test will have to decrease to be available to a wider range of patients.

Unexpectedly, 30/81 noninfectious patients (~40%) had false-positive (FP) results, of whom 24 had viruses with a low RPM (1 to 490), including CMV, HHV-6B, EBV, HSV-1, etc. Although it has been reported that the microbes detected in CSF specimens of healthy people are probably from exogenous contamination, such as normal saline, DNA extraction buffer, and related tissues (skin, muscle, and blood vessels) during lumbar puncture (41), it is still difficult to collect muscle and blood specimens during the collection of CSF specimens from large numbers of people, especially healthy people. Thus, it remains unclear whether the microbes detected in our noninfectious patients presented latent infections in immune cells or not. Despite the high sensitivity of cfDNA mNGS of trace microbes, viruses, and parasites, clinicians should perform strict disinfection measures before puncture and construct a colonizing microorganism database to filter out noise signals and reduce false-positive results.

In our study, mNGS tests were performed using cfDNA and wcDNA without host depletion to evaluate the performance of extraction methods in diagnosing pediatric CNS infections. Previous reports showed that both the detection rate (29) and TCR (21) of wcDNA mNGS using CSF samples (42) were only approximately 50%, consistent with our data. As reported, the lysis of the cell wall in wcDNA extraction could increase the risk of DNA degradation and bring in contamination of engineered strains from reagents (20, 27), which can significantly influence pathogen DNA recovery, especially for trace pathogens such as viruses and mycobacteria (27, 42–44). On the other hand, the sensitivity of mNGS was determined by the pathogen DNA ratio in the sample. The extraction of wcDNA without host depletion can increase the release of human DNA (20). The cfDNA was directly extracted from the low-cellularity supernatant, resulting in the pathogen DNA ratio of cfDNA being higher than that of wcDNA in the same sample (28). The above might explain why the sensitivity and TCR of cfDNA mNGS were higher than those of wcDNA mNGS. One unexpected finding was that wcDNA mNGS had a higher specificity and positive predictive value (PPV). This result could be explained by the fact that the viruses were detected by cfDNA mNGS in all the FP cases, but patients were asymptomatic, possibly due to the latent status of the viruses. To our knowledge, this is the first study to evaluate the different mNGS methodologies in CSF samples from children, and mNGS using CSF cfDNA may be a preferred examination in the diagnosis of pediatric CNS infections.

In-depth etiological analysis showed that the distribution of pathogenic bacteria and viruses detected was basically consistent with the epidemiology of pathogens in pediatric CNS infections (45). The development of the human immune system is a continuous process, potentially gaining peak function during adolescence (46), which makes young children at greater risk of developing infections than older children and adults and makes them more vulnerable to disseminated infections (47, 48), including CNS infections. Consistently, we found that infants had the highest abundance of pathogenic species and rates of meningitis secondary to H. influenzae, and S. pneumoniae was highest among children <5 years of age (49–51). Meanwhile, S. pneumoniae was the main causative pathogen identified in all three age categories, and H. influenzae was only identified in infants and preschool children, which was in accordance with a previous Chinese report from Yunnan (52) and other studies from Sweden (53), Brazil (54), and England and Wales (55) but differed from data collected in mainland China (56). Most children and adolescents with viral CNS infections can recover completely in ~7 to 10 days without specific treatments (9). However, the prognosis depends on the patient’s age and the pathogen (57); for example, the mortality rates of encephalitis caused by HSV reach 70% in untreated children, and long-term sequelae are common (~30%) in treated pediatric patients (58). CNS infection caused by CMV may be a severe complication in immunocompromised children, with high rates of morbidity and mortality (9). In fact, in a previous large study, researchers observed that 91.4% of children with viral meningitis or encephalitis were immunocompetent and had no reported underlying conditions (59). HSV and CMV were the main causative pathogens involved in our patients with viral infections (89.1% were previously healthy), but only 4 children had sequelae, such as epilepsy and cognitive impairment. EVs can be detected in 85% of meningitis cases in children, while we did not identify RNA viruses, including EVs, by conventional methods (PCR and viral serologic tests). Thus, we did not perform RNA mNGS to validate the potential RNA viruses in this study. In addition, viruses were detected in more than 33 bacterial infections, and studies have shown that viruses and bacteria can exploit cross-kingdom interactions to their mutual benefit, which may lead to an increase in both the susceptibility to secondary bacterial infections and the severity of the bacterial infections (60, 61). Although studies remain somewhat limited, these findings suggest that viruses might establish a latent infection and that additional antiviral therapy needs to be considered in some respects. Hence, children cannot simply be treated as “little adults” but rather need pediatric-specific treatment strategies that should be stratified by age to cover the most likely organisms.

Researchers have pointed out that mNGS results are not influenced by prior antibiotic administration (62). However, PCR of mNGS in our study changed with the duration of empirical therapy, which was consistent with a published study (29). Tomasz et al. (63) pointed out that empirical therapy can cause lysis in the cell wall of some pathogens or directly kill some pathogens, which might explain why the concentrations of cfDNA and PCR of mNGS increased in CSF in a short time. Our data also showed a significant difference between the TP and FN groups in the sampling-medication time of children with bacterial CNS infections. However, this finding was contrary to those from previous studies, which suggested that mNGS results were not affected by empirical treatment in bacterial CNS infections (56). The positive rate of mNGS showed a downward trend from 4 days after empirical therapy in our study, because long-term and continuous antimicrobial therapy inhibited the growth of pathogens. Consistently, Zhang et al. (29) demonstrated that anti-infection treatment of >4 days on adults with CNS infections significantly influenced the detection rate of mNGS. Hence, the dynamic process suggested that mNGS should be conducted at the right time to improve the efficiency.

Several limitations need to be noted regarding the present study. First, the research was retrospective and was limited by the single-center nature of the study, with a relatively small population of subjects. The CSF microbiome data were not obtained from a healthy population as a baseline; therefore, it was difficult to generate an optimal threshold for pathogen identification. Our results need further validation in larger-scale clinical trials. Second, due to the preference of subjects with DNA pathogenic infections and constraints of laboratory conditions, our current mNGS test targeted only DNA, and future efforts should be made to incorporate neuroinvasive RNA virus detection. Third, pathogens detected by mNGS were not validated with an additional molecular method at the genetic level. More samples from multiple hospitals are needed to evaluate the performance between mNGS of cfDNA and wcDNA and to orthogonally validate the infections identified by mNGS. Finally, the ability of cfDNA mNGS to help with the timely adjustment of treatments should also be evaluated in the future.

### Conclusion.

Our study emphasized that mNGS had overall superiority to conventional methods on causative pathogen detection in CNS-infected pediatric patients, and it was even better than wcDNA mNGS. The best timing for cfDNA mNGS detection ranged from 1 to 6 days after the start of empirical anti-infection therapy, but pediatric-specific treatment strategies need to be tailored to their pathogen profiles at different ages. The earlier mNGS is started, the better bacterial CNS infections can be identified. This research needs to be further validated in large-scale clinical trials to improve the clinical applications of cfDNA mNGS.

## MATERIALS AND METHODS

### Trial design and oversight.

We retrospectively studied a cohort of 338 children with definite clinical diagnoses, including 257 patients with CNS infections and 81 patients with noninfectious neurological disorders, who were admitted to the Department of Pediatric Neurology, Xi’an Children’s Hospital, between January 2019 and September 2021. The inclusion criteria were in line with guidelines from the International Encephalitis Consortium and SCMID Study Group for Infections of the Brain (29, 64, 65). The enrollment criteria of final diagnosis of CNS infection were as follows: (i) patients with altered mental status (decrease or change in awareness level or personality change lasting at least 24 h) and with at least 2 of the characteristics of fever (body temperature over 38.2°C within 72 h before and after the onset of symptoms), seizures (confirmed by clinical symptoms or EEG), newly occurring focal neurological deficits, elevated numbers of cells in CSF (white blood cell count greater than 5 cells/mm^3^), and EEG or neuroimaging consistent with encephalitis changes; and (iii) at least one etiology criterion should be met, i.e., (a) a positive CSF culture of pathogenic microbes, (b) a specific positive result by polymerase chain reaction, specific antibody test, or pathological examination. Noncentral nervous system infection was defined as clinical characteristics that did not meet the above clinical criteria. The exclusion criteria were as follows: (i) age ≤28 days or >18 years; (ii) not undergoing lumbar puncture and CSF examination or with incomplete clinical data; and (iii) intracranial infection caused by trauma or surgery.

Conventional microbiological methods, such as smear, culture, serologic tests, enyme-linked immunosorbent assay (ELISA), the tuberculin skin test/ (T-spot), TB test, and so on, were performed according to clinical necessity. Viral ELISA can detect specific IgM and IgG of 13 common viruses including norovirus, human gammaherpesvirus 4 (EBV), human betaherpesvirus 5 (CMV), human alphaherpesvirus (HSV), human orthopneumovirus (RSV), adenovirus, influenza A virus, influenza B virus, enteroviruses, echoviruses, etc. Microbes reported by the clinical laboratory as representing laboratory, skin, or environmental contaminants were not included. Clinical data of all patients, including baseline demographic characteristics, chronic illnesses and disabilities, laboratory test results, clinical diagnosis, antibiotic administration, and prognosis, were collected and subjected to subsequent statistical analysis. Meanwhile, we established age categories as follows: infants (0 to 1 years), preschool children (1 to 6 years), and school-aged children (>6 years), according to the development of the human immune system (46).

### Ethics statement.

The research was approved by the Ethical Review Committee of Xi’an Children’s Hospital, Xi'an, China (approval number 20220023). Written consent was obtained from the subjects or their guardians.

### DNA extraction and metagenomic next-generation sequencing.

Approximately 1 to 2 mL of CSF from each subject was shipped on dry ice to Hugobiotech Co., Ltd. (Beijing, China) to perform mNGS detection. According to the manufacturer's instructions for the QIAamp DNA Micro kit (Qiagen, Hilden, Germany), cfDNA and wcDNA were extracted from CSF samples. For cfDNA extraction, the cells from CSF samples were removed through centrifugation at 1,600 × *g* for 10 min, followed by centrifugation at 16,000 × *g* for 10 min at 4°C. cfDNA was extracted and purified from the supernatant. For wcDNA extraction, CSF samples were directly homogenized using a TGrinder H24R grinding instrument (Tiangen, Beijing, China). Then, the treated specimens were centrifuged at 10,000 × *g* for 5 min, and the supernatant was used for wcDNA extraction using a QIAamp DNA Micro kit (Qiagen, Hilden, Germany). In this study, the extractions of cfDNA and wcDNA were performed in 338 and 124 samples, respectively. The quality and concentration of DNA samples were monitored by a Qubit fluorometer (Thermo Fisher Scientific, MA, USA), and metagenomics libraries were constructed by a QIAseq Ultralow input library kit (Qiagen, Hilden, Germany). Library quality control was performed with a Qubit fluorometer (Thermo Fisher Scientific, MA, USA) and Agilent 2100 Bioanalyzer (Agilent Technologies, Palo Alto, CA, USA). The qualified libraries with different barcode labeling were pooled and sequenced on an Illumina Nextseq 550 platform (Illumina, San Diego, CA, USA). In parallel with the clinical samples, positive controls and negative controls (including a nontemplate control [NTC]) were also set for mNGS detection with the same procedure and bioinformatics analysis. The NTC samples enabled estimation of the number of background reads except for each taxon (46). High-quality data were generated after filtering out adapter, low-quality, low-complexity, and shorter reads (17). Next, human reads were removed by mapping reads to the human reference genome (GRCh38) using Bowtie2 (66). The remaining data were aligned to the microbial genome database (https://ftp.ncbi.nlm.nih.gov/genomes/) using Burrows-Wheeler alignment (67). The read number and RPM of each detected pathogen were calculated, and the microbial composition was determined. The formula for calculating RPM was as follows: RPM of pathogen = (number of reads mapped to the pathogen × 10^6^)/(total number of mapped reads from given library).

We developed an NTC-based strategy to filter reagents and prevent laboratory contamination (68). Further details on sequencing and reporting methods are provided in Text S1 in the supplemental material.

### Diagnostic assessment.

The researchers performing mNGS were blinded to the diagnosis that was made on the basis of conventional methods and clinical symptoms, and the mNGS results were evaluated by at least two experienced physicians. For the infectious diagnosis group, mNGS-positive and mNGS-negative results were classified into true-positive (TP) and false-negative (FN) results, respectively. For the noninfectious diagnosis group, we defined the mNGS-positive and mNGS-negative results as false-positive (FP) and true-negative (TN) results, respectively. The sensitivity, specificity, PPV, and negative predictive value of different methods were compared in pairs. The total coincidence rates (TCRs) of mNGS and conventional tests were evaluated based on clinical diagnosis.

### Statistical analyses.

SPSS 22.0 statistical software was applied for data processing, and measurement data were expressed as the mean ± standard deviation. The count data were expressed as cases or percentages, and a *t* test was used for the comparisons between two groups. The chi-square test or Fisher’s exact test was used to evaluate independent binomial variables, and a *P* value of <0.05 was considered statistically significant.

### Data availability.

The datasets used and/or analyzed during the current study are available at National Genomics Data Center (http://ngdc.cncb.ac.cn), reference number PRJCA009189.
