# Supplementary material for: Diagnosis of Neurological Infections in Pediatric Patients from Cell-Free DNA Specimens by Using Metagenomic Next-Generation Sequencing
Source: Microbiol Spectr. 2023 Jan 18;11(1):e02530-22. doi: 10.1128/spectrum.02530-22 (PMC9927296; doi:10.1128/spectrum.02530-22)
Supplement: Supplemental file 1 — Supplemental material. Download spectrum.02530-22-s0001.pdf, PDF file, 0.4 MB [file spectrum.02530-22-s0001.pdf]

Supplementary Online Content

Diagnosis of Neurological Infections in Pediatric Patients from Cell-Free DNA Specimens using Metagenomic Next-generation Sequencing

Xia Li[1#], Le Yang[1#], Dongjing Li[1#], Xuying Yang[2], Zhijing Wang[1], Mengyi Chen[1], Fang Wu[1], Xiangjun Dou[1], Mengmeng Niu[1], HongYan Qi[1], Ting Deng[1], Han Xia[2*], Dong Wang[1*]

[1]Department of Pediatric neurology, Xi'an Children's hospital, No. 69, Xijuyuan Lane, Lianhu District, Xi'an City, Shaanxi Province, China, 710003.
[2]Department of Scientific Affaires, Hugobiotech Co., Ltd., No.1, East Disheng Road, Beijing, China, 100176

[#]Xia Li, Le Yang, and Dongjing Li contributed equally to this work.

* Correspondence: Dong Wang, WDhope20@163.com; Han Xia, xiahan@hugobiotech.com

eMethods

eFigure 1. Contingency tables for the clinical diagnosis with cfDNA mNGS and conventional methods sets, respectively.

eTable 1. The comparison between the true positive (TP) and false negative (FP) mNGS results.

eReferences

This supplementary material has been provided by the authors to give readers additional information about their work.

eMethods

Cell-free DNA Extraction

CSF samples were collected through centrifugation at 1600×g for 10 min, followed by centrifugation at 16,000 ×g for 10 min at 4℃. According to the manufacture's instruction for the QIAamp DNA Micro Kit (QIAGEN, Hilden, Germany), cfDNA was extracted and purified. The quality and concentration of DNA samples were monitored by a Qubit Fluorometer (Thermo Fisher Scientific, MA, USA).

Whole-cell DNA Extraction

The 124 CSF samples were homogenized using grinding instrument, TGrinder H24R (TIANGEN, Beijing, China) and centrifuged at 10,000g for 5 min. The supernatant was used for wcDNA extraction according to the manufacture's instruction for the QIAamp DNA Micro Kit (QIAGEN, Hilden, Germany). The quality and concentration of DNA samples were also monitored by a Qubit Fluorometer (Thermo Fisher Scientific, MA, USA).

Metagenomic next-generation sequencing and analysis

Metagenomics libraries were constructed by QIAseq Ultralow Input Library Kit (QIAGEN, Hilden, Germany) according to its manual. The qualified library was sequenced on Nextseq 550 platform (Illumina, San Diego, USA). In parallel with the clinical samples, positive control and negative control (non- template control, NTC) were also set for mNGS detection with the same procedure and bioinformatics analysis.

After filtering out adapter, low-quality, low-complexity, and shorter reads of <35bp(1), high-quality sequencing data were generated. Next, human reads were removed by mapping reads to human reference genome (GRCh38) using Bowtie2(2). The remaining clean data was aligned to the microbial genome database (ftp://ftp.ncbi.nlm.nih.gov/genomes/) using Burrow-Wheeler Aligner software(3). The reads number and reads per million (RPM) of each detected pathogen was calculated and the microbial composition was presented. The positive criteria for the mNGS result were set as follows:

1) For the detected bacteria (*Mycobacterium* excluded), fungi (*Cryptococcus* excluded), and parasites: a) genome coverage of the unique reads mapped to this microorganism ranked top10 of the same kind of microbes and the microorganism was not detected in the NTC; or b) RPMsample/RPMNTC was > 10 (RPMNTC≠0).

2) For viruses, *M. tuberculosis*, and *Cryptococcus*: a) the unique reads of this microbe was not detected in NTC but at least 1 specific read was mapped to species; or b) RPMsample/RPMNTC was > 5 (RPMNTC≠0).

After the prior analysis, if mNGS results were in accordance with the patient’s clinical features, the detected pathogens would be considered as causative agents, or the detected organisms would be classified as non-pathogenic microbes(4).

cfDNA mNGS	Conventional methods +	Conventional methods -
+	33	212
-	7	94

Sensitivity：82.5%

Specificity：30.7%

PPV：13.5%

NPV：93.1%

eFigure 1. Contingency tables for the clinical diagnosis with cfDNA mNGS and conventional methods sets, respectively

eTable 1. The comparison between the true positive (TP) and false negative (FP) mNGS results.

		**True positive (TP)**	**False negative (FN)**	**P value**
**Total** (TP, n=177; FN, n=78)	Days of onset	12.3±14.9	17.1±19.2	0.0052
	Days of mNGS detection after admission	3.6±3.9	4.4±6.4	0.1321
	Days of antibiotic usage	5.9±7.5	9.1±10.5	0.0232
**Bacteria** (TP, n=102; FN, n=29)	Days of onset	10.0±10.5	17.0±14.6	0.0153
	Days of mNGS detection after admission	3.5±3.5	6.1±9.7	0.0415
	Days of antibiotic usage	6.0±7.5	13.0±13.6	0.0064
**Viruses** (TP, n=67; FN, n=42）	Days of onset	14.3±18.8	16.5±23.1	0.6399
	Days of mNGS detection after admission	3.6±4.0	3.1±2.5	0.9576
	Days of antibiotic usage	4.8±5.7	5.4±5.4	0.3508

eReference

1. Chen Y, Feng W, Ye K, Guo L, Xia H, Guan Y, Chai L, Shi W, Zhai C, Wang J, Yan X, Wang Q, Zhang Q, Li C, Liu P, Li M. 2021. Application of Metagenomic Next-Generation Sequencing in the Diagnosis of Pulmonary Infectious Pathogens From Bronchoalveolar Lavage Samples. Front Cell Infect Microbiol 11:541092.
2. Langmead B, Salzberg SL. 2012. Fast gapped-read alignment with Bowtie 2. Nat Methods 9:357-9.
3. Li H, Durbin R. 2009. Fast and accurate short read alignment with Burrows-Wheeler transform. Bioinformatics 25:1754-60.
4. Zhang Y, Cui P, Zhang HC, Wu HL, Ye MZ, Zhu YM, Ai JW, Zhang WH. 2020. Clinical application and evaluation of metagenomic next-generation sequencing in suspected adult central nervous system infection. J Transl Med 18:199.
